# Results from Survey to Assess Current Trends in Surgical Practice in the Management of Women with Early Stage Cervical Cancer within the BGCS Community with an Emphasis on Routine Frozen Section Examination

**DOI:** 10.1155/2017/2962450

**Published:** 2017-07-17

**Authors:** Kumar Gubbala, Alexandros Laios, Thulumuru Kavitha Madhuri, Pubudu Pathiraja, Krishnayan Haldar, Sean Kehoe

**Affiliations:** ^1^Gynaecologic Oncology Unit, Churchill Hospital, Oxford University Hospitals, Oxford, UK; ^2^Department of Gynaecological Oncology, The Royal Surrey County Hospital NHS Foundation Trust, Guildford, UK; ^3^Faculty of Health and Medical Sciences, University of Surrey, Guildford, UK; ^4^School of Cancer Sciences, University of Birmingham, Birmingham, UK

## Abstract

In the UK, more than 3,200 new cases of cervical cancer are diagnosed each year. Early stage cervical cancer (IA2-IB1) treatment comprises central surgery mainly in the form of radical hysterectomy or fertility sparing surgery including trachelectomy as well as systematic pelvic lymphadenectomy to detect metastases and adjust treatment accordingly. Given the variation in determining the lymph node (LN) status, a major prognosticator, we reviewed the current UK practice of LN assessment in women undergoing surgery for early cervical cancer. A 7-question, web-based survey, screened by the BGCS committee, was circulated amongst BGCS members. The overall response rate was 51%. Only 12.5% of the respondents routinely performed frozen section examination (FSE); the main reasons for not doing FSE were the pressure on theatre time (54.5%) and the lack of available facilities (48.5%). When positive pelvic nodal disease was detected, in 21 out of 50 (42%) the planned radical hysterectomy (RH) was aborted. More than 70% of the respondents routinely performed RH without any prior resort to pelvic lymphadenectomy. Pretreatment surgical para-aortic LN assessment was performed by 20% of the respondents. The survey confirms the diversity of the UK practice patterns in the surgical treatment of early cervical cancer.

## 1. Introduction

Cervical cancer is the fourth most common cancer in women accounting for nearly 8% of all female cancer deaths [1]. In the UK, more than 3,200 new cases are diagnosed annually but more than 80% survive their disease for more than one year. The reduction in cervical cancer mortality is considered a major triumph of an established national screening program. Early stage cervical cancer (IA2-IB1) treatment comprises two steps: central surgery mainly in the form of radical hysterectomy (RH) or fertility sparing surgery including trachelectomy as well as systematic pelvic lymphadenectomy (PLND) to detect metastases and adjust treatment accordingly. Nevertheless, if surgery is not an option, an alternative is chemoradiation (CRT), which affords similar survival rates [2]. Where surgery is likely to be the single therapeutic modality, this must be deemed the optimal approach based on the duration of intervention, recovery time, and survival benefit.

In early stage cervical cancer, pelvic lymph node (PELN) and para-aortic lymph node (PALN) status remain strong prognostic indicators, albeit interestingly not included in FIGO staging, which is based on clinical and imaging evaluation to establish equity of care worldwide [3]. Equally, in early stage disease, surgery would be considered the most likely course of care. However, if nodal disease is detected in histological specimens after surgery, patients would be exposed to adjuvant CRT, thus significantly increasing morbidity. Predicting Lymph node (LN) status would allow for proceeding directly to CRT obviating the need for and delays in surgery reducing the morbidity [3].

In recent years, strict LN mapping algorithms including sentinel lymph node (SLN) biopsy have been safely applied to the management of early stage cervical cancer [4]. One such option to possibly minimise morbidity with both surgical and CRT modalities is the use of intraoperative frozen section examination (FSE) of all the LNs [5]. In this era of stringent economical barriers, we set out a survey to ascertain the use of FSE of PELNs in early stage cervical cancer and inform of the potential variation in practice within the British Gynaecological Cancer Society (BGCS).

The aim of this survey was twofold:

- To review the current UK practice and gather information on any possible variation
- To contribute towards the development of agreed standards.

The authors were particularly interested in the percentage of clinicians who routinely (1) abandoned RH in the advent of positive FSE, (2) performed RH without any previous resort to PLND, and (3) carried out pretreatment surgical PALN assessment, when indicated.

## 2. Materials and Methods

A questionnaire was designed and completed via the administration of an anonymous, nonvalidated, commercially available online survey (SurveyMonkey®). Following regular workshops, a 7-question survey was formulated by a group of gynaecological oncology specialists working in the same institution (AL, KG, PP, and KH). The questionnaire was created to assess the early stage cervical cancer management when FSE of all PELNs was indicated. It did not differentiate amongst cancer substages. It was screened and reviewed by the BGCS committee in May 2015 and access was granted to the membership for purposes of circulation of the survey. The survey was sent to members with working email addresses in 2 rounds over a 6-week period. The email invitation contained a survey scope and a link to a website access to the survey (https://www.surveymonkey.com/s/G7YBX78). The respondents were stratified based on years of experience and the numbers of new cases of cervical cancer surgically treated per annum.

Data collection and analysis were carried out using the SurveyMonkey database. Questions within the survey allowed for one answer (yes/no) to be selected. For some questions, the option of additional comments was additionally provided. Descriptive statistics were used to summarise the results of the questionnaire survey. Percentages were calculated based on the number of responses per individual question and not the total number of survey participants. Fisher's exact test was used to check for statistical significance, set at *p*: 0.05.

## 3. Results

A total of 117 emails were sent out; however 19 email addresses were not valid at the time of the survey. A total of 50 responses were collected out of 98 potential respondents for an overall response rate of 51%. The demographic characteristics of the respondents are shown in Table 1. All 50 respondents (100%) answered the question “years in practice.” Forty-eight out of 50 (98%) members responded to the question “number of new cases of cervical cancer treated surgically per year.” Most of the respondents had been in practice for over 10 years operating on 10–30 new patients annually. 

The 7 survey questions addressing the practice patterns of gynaecological oncologists with their response rate are displayed in Table 2. To identify pelvic nodal metastases, only 6 out of 48 (12.5%) routinely performed FSE of all PELNs (question 1, Table 2). When positive pelvic nodal disease was detected, 21 out of 50 (42%) respondents abandoned the planned RH (question 2, Table 2). A small fraction, 4 out of 48 (8.6%) respondents, performed a two-stage procedure including PLND first, followed by RH at a later stage (question 3, Table 2). Thirty-two out of 45 (71.1%) respondents routinely performed RH with PLND, as a one-stage procedure without any prior surgical LN assessment (question 4, Table 2). This practice appeared specific for 1b2 tumours. A small percentage of respondents (28.9%) did not complete RH due to suspicious LNs at preoperative magnetic resonance imaging (MRI) or unexpectedly at the time of surgery. The majority (56.25%) concurred that intraoperative findings of LN disease alter management (question 5, Table 2).

With respect to the PALNs, the commonest assessment modalities employed, when indicated, included CT/MRI scan (83.3%), positron emission tomography (PET) scan (39.5%), and intraoperative PALN sampling (14.6%) followed by systematic lymphadenectomy (6.25%) (question 6, Table 2).

Reasons given for not doing routine FSE of all resected LNs included the pressure on theatre time (54.5%), the lack of available facilities (48.5%) and the absence of evidence that routine FSE could alter management (33.3%), the justification for CRT based on other parameters (21.2%), and the preference for a two-stage procedure (6%) (question 7, Table 2). No statistical significance was observed between responses and demographic variables.

## 4. Discussion

The surgical management of early stage cervical cancer is an important area of debate. FSE for nodal evaluation has the potential to minimise bimodal therapy (surgery and CRT) by detecting metastatic disease. A recent study suggested that use of this approach could keep the rate of bimodal treatment to 10% [6]. The survey represents the first attempt to specifically address the current UK gynaecological practice on the use of intraoperative FSE with respect to the LNs. We aimed to assess this practice in the absence of consensus and then answer why most of the respondents do not practice FSE, which may reflect the putative idea that the procedure adds little to the management of this cohort of cancer patients.

The survey was screened and ratified by the BGCS committee. We surveyed the BGCS members to identify the most common practice inclusive of current preferences and opinions in the diagnosis and surgical treatment of early stage cervical cancer in the UK. To our knowledge, this is the only UK published survey on the topic. To ascertain individual gynaecological oncologist practice, the questionnaire was targeted to this group rather than sent to specific gynaecological cancer centres. The most important finding was the paucity of FSE use in practice, the main reasons being the pressure on theatre time and the lack of available facilities. Considering the potential of FSE to reduce unnecessary surgery and morbidity, this finding is of some concern. The lack of evidence is a reasonable response to not using FSE, although, in the UK, the low level of FSE use at present would hinder the development of appropriate studies. This finding does not essentially reflect a low rate of performing laparoscopic PLND for surgical staging of the disease. In the UK, minimally invasive surgery has been widely accepted in the surgical management of cervical cancer in compliance with the international community with comparable oncological and surgical outcomes to open surgery, including LN yield [7].

In our institution, the authors routinely perform FSE with standard pathologic evaluation of all retrieved PELNs and not just for any enlarged or suspicious nodes as per preoperative imaging. The setting involves a dedicated gynaecological pathologist on site to perform and report on FSE. The authors have recently published on the value of FSE as a diagnostic test, which reached a sensitivity of 86.7% and a specificity of 100% [8]. These rates are comparable to those reported in the literature [9, 10]. In our cohort, the only false negative cases contained micrometastases in the PELNs, which were not identified at FSE. Therefore, this strategy does indeed alter our intraoperative management. In addition, a low risk group of patients at least risk of nodal disease, which may be spared of FSE, was identified [8].

FSE of sentinel lymph nodes (SLNs) can also accurately predict the status of PELNs in early stage cervical cancer with the sensitivity of bilateral SLN ultrastaging reaching 97% [11]. The concept of SLN biopsy is gaining much popularity in current practice, yet not widely adopted for cervical cancer in the UK. Sensitivity of SLN ultrastaging is high for the presence of both micro- and macrometastases even in the case of non-SLN PELNs [12]. Nevertheless, assessing all the LNs by use of an ultrastaging protocol could not be applicable to all patients due to the financial cost. Detection of micrometastases with the time constraints afforded by FSE inevitably influences its accuracy [13]. Nevertheless, in cervical cancer, metastatic disease less than 5 mm does not appear to impact survival [14].

Introducing universally accepted standardised FSE protocols and ensuring reporting by specialist pathologists have been shown to improve FSE accuracy [15]. Provided that FSE accuracy is satisfactory, identification of patients at low risk for nodal metastases may theoretically help inform selected patients in the absence of enlarged lymph nodes or SLNs towards less aggressive surgical interventions and reduce the lymphadenectomy-related morbidity [16]. Implementation of FSE with ultrastaging may prove cost-effective in high risk patients with respect to avoiding morbidity and should be explored.

In our survey, most of the respondents did not routinely perform FSE, which reflects the tradition of UK practice [17]. Inefficient use of valuable theatre time appeared to be the main reason, followed by expressed concerns regarding the difficulty of examining all nodal tissue by FSE [18]. Whether such decision was influenced by preoperative imaging findings would not be possible to conclude as it was not addressed in the questionnaire. A cost analysis of routine FSE has never been performed in cervical cancer, although it positively influenced management in other cancer types [19].

It is interesting to note that if positive LNs were encountered, only 42% of the respondents would abort the planned RH, possibly because these respondents did not make use of FSE. There is no evidence that proceeding affords any benefit; indeed, the contrary is known and the use of brachytherapy can be more difficult when the uterus and cervix are removed. The questionnaire was not developed to scrutinise this element of care, but a further study on this issue would be useful. However, controversy exists as to whether RH should be abandoned if LN metastasis was detected at the time of surgery [5]. This hesitance may be triggered by a perception for higher morbidity rates when surgical treatments are compared to CRT [2] or knowledge that surgical morbidities may be associated with the radical parametrectomy rather than the PLND [4]. To add more to the controversy, a recent study suggested that completing rather than abandoning RH during intraoperative detection of positive LNs might result in a better pelvic control. However, this was subjected to selection bias and if corrected for LN variables, there was no improvement on disease-free survival [20].

Thirty out of 45 respondents routinely performed a RH with PLND, without resort to prior systemic lymphadenectomy or FSE. Such an approach should take into consideration RH-related morbidity, when adjuvant therapy is indicated, given that equivalent 5-year survival was observed between the completed and abandoned RH groups in a recent study [21]. A two-stage procedure including PLND followed by RH was not favoured by the vast majority of the respondents (91.6%) in the absence of large tumours (>4 cm) or adverse pathological features. Additional surgical times would encompass increased costs, potential additional morbidity, and negative emotional impact on the patient.

Another point of controversy is whether pretreatment surgical PALN assessment—when indicated, as in stages beyond 1A2—offers survival benefits and if so, whether the associated morbidity outweighs the risks. A recent Cochrane review concluded that there was insufficient evidence to support the benefit of surgical PALN assessment [22]. Hence, it should be restricted to cases where the suspicion of disease presence is high to avoid undertreatment. In fact, PALN involvement without positive PELNs is rare and the yield of routine PALN surgery in early cervical cancer is low [23]. Not surprisingly, most of the respondents would prefer accurate, noninvasive presurgical diagnostic tests to determine nodal status and thus triage care appropriately. Currently, CT/MRI and PET/CT are employed. In fact, PET/CT is the most reliable imaging modality to assess extrapelvic disease. Since the true-positive rate of PET is high, the only indication for staging surgery that could be considered is the rarity of isolated para-aortic uptake with no uptake in the pelvic region to avoid mismanagement due to a false-positive result, allowing for extension of radiation therapy fields to include the para-aortic area. [22]. For patients with positive PELNs, PALN involvement rate is not negligible and can be as high as 50% [24]. False negative results in the para-aortic region have been recorded in 12% of patients, rising to 22% in those with uptake during PET of the PELNs [25]. The proportion of positive PELNs with uptake on PET-CT at the time of lymphadenectomy is higher than the false negative rate. Surgical staging surgery should, therefore, be mandatory when no uptake is recorded in PALNs on PET-CT to avoid undertreatment. By contrast, the proportion of PELNs with no uptake on PET-CT at the time of lymphadenectomy is lower than the false negative rate. PALN staging surgery could be indicated in these cases, but then only if potential morbidity is low [25]. The effect on survival of potential delay of chemoradiation owing to use of PET and staging surgery has not been addressed by RCTs. ESGO and BGCS guidelines, in addition to the current algorithms for the management of cervical cancer, are expected to be released soon and shed light to this important debate.

Inevitably, there are limitations to any survey, the obvious being the response rate. Above 50% this is not disastrous, but a response closer to 60% would have been preferable. As BGCS members could still represent a variety of disciplines including surgeons but also clinical and medical oncologists, it is likely that, for some members, the survey was not found relevant to their clinical practice. As the questionnaire was sent blindly, it was difficult to differentiate between those respondents who were gynaecological oncology surgeons and those who were not. On this note, it was also not possible to assume the repartition amongst surgeons, medical, and radiation oncologists with respect to their answers.

Potential biases include the fact that those individuals who respond because they have FSE available are thus advocates, but other respondents are from those strongly opposed to FSE. These biases are difficult to overcome and must be recognised. Equally, imperfection may exist in the questions as they are not specific to early cervical cancer substages where some management options would not be justified. In that respect, the authors are uncertain whether fertility sparing surgery would impact FSE use. Conclusions regarding the relevance of the anatomical site or the numbers of positive LNs with intraoperative decision-making could not be made. How the respondent interprets the question may be another limitation. Indeed, not all respondents answered all questions. The survey was confined to individual practice but some reflectance of eccentric practice could not be avoided. An attempt to minimise response biases by addressing direct questions and facilitating additional comments in a free text format should be credited. The questionnaire was screened and validated by the BGCS committee; it was circulated in 2 rounds over a substantial time frame to increase response rate.

## 5. Conclusions

This is the first survey to ascertain the role of FSE in the surgical management of early cervical cancer with a special emphasis on FSE of all PELNs, which illustrates substantial diversity in the UK practice. Therefore, this remains the only evidence available on clinical practice in this specific disease entity. It is interesting to note the spectrum in the accessibility of modern FSE techniques, and, more importantly, the variation in practice when positive nodal disease is encountered. A National debate is required, as from a patient perspective it may seem unusual that surgical practice regarding RH in the face of positive nodes differs to such an extent.

## Figures and Tables

**Table 1:** Demographics of survey participants with their response rates.

Variable	Number	Response rate (%)^*¥*^
Years in practice		
Less than 5 years	10	20.0
5–10 years	8	16.0
More than 10 years	32	64.0

Number of new cases of cervical cancer treated surgically per year		
Less than 10/year	20	41.6
10–30/year	23	48.0
31–50/year	5	10.4
More than 50/year	0	0.0

^*¥*^Percentages were calculated based on the number of responses per individual question and not the total number of survey participants.

**Table 2:** Questionnaire to British Gynaecological Cancer Society (BGCS) members.

Question		Response rate (%)
Q1	Do you routinely perform an intra-operative frozen section evaluation of all lymph nodes prior to proceeding to a radical hysterectomy ? (Y/N)	48.0 (96%)
Q2	Do you complete radical hysterectomy following positive frozen section examination? (Y/N/Do not do frozen section examination)	50.0 (100%)
Q3	Do you perform a two-stage procedure, i.e, lymphadenectomy first followed by radical hysterectomy at a later stage depending on the results? (Y/N)	48.0 (96%)
Q4	Do you always perform the hysterectomy without any prior surgical assessment of pelvic lymph nodes intra-operatively by frozen section or pre-operatively by a separate lymphadenectomy ? (Y/N)	45.0 (90%)
Q5	Does management become altered in all cases with positive lymph nodes on frozen section examination? (Y/N/Other)	48.0 (96%)
Q6	How do you assess para aortic lymph nodes?	40.0 (80%)
Q7	If you do not routinely perform frozen section examination of all lymph nodes, please explain why	33.0 (66%)
